# The genetic technologies questionnaire in the Greek-speaking population: the moral judgement of the lay public

**DOI:** 10.3389/fgene.2025.1594724

**Published:** 2025-05-13

**Authors:** Florian Melchior, Ioanna Antigoni Angelidou, Maria Chorianopoulou, Birgit Teichmann

**Affiliations:** ^1^ Network Aging Research (NAR), Heidelberg University, Heidelberg, Germany; ^2^ Department of Philosophy, National and Kapodistrian University of Athens, Athens, Greece

**Keywords:** genetic technologies, genome editing, genetic testing, ethics, knowledge, moral status, autonomy, religiosity

## Abstract

**Introduction:**

Advancements in life sciences have significantly boosted biomedical capabilities. Genetic testing forecasts hereditary traits and disease susceptibility, while CRISPR/Cas allows permanent genome alterations. However, ethical considerations arise regarding the morality of these capabilities, particularly concerning the moral status, autonomy, and privacy of living beings. The lack of valid instruments to assess moral judgment in genetic technologies highlights the need for this study, aiming to translate and validate the “Genetic Technologies Questionnaire” (GTQ) and the short version of the “Conventional Technologies Questionnaire” (CTQ5) into Greek. As the full version of the GTQ with 30 questions could be too extensive for some studies, we also tested other versions: The short versions GTQ20-GR and GTQ5-GR which were already presented in the original study, as well as a version which included questions solely about humans (GTQ-H-GR) and is intended for use in human research and therapy, and the GTQ-Moral Status (GTQ-MS-GR), which included questions about genetic testing and gene editing in different living beings to investigate differences in moral status.

**Methods:**

A cross-sectional study involved 250 participants who completed an online questionnaire, assessing internal consistency, structural validity, known-groups validity, floor/ceiling effects, and retest reliability (subset of 50 participants). Correlational analyses explored relationships with education, age, genetic knowledge, religiosity, and genetic testing experience. The study followed the STROBE checklist for reporting.

**Results:**

The GTQ-GR (Cronbach’s α = 0.929) and GTQ20-GR (α = 0.935) exhibit high reliability and stability in assessing moral judgment among lay people, whereas the GTQ5-GR (α = 0.866) and CTQ5-GR (α = 0.758) displayed some weaknesses. Participants tended to rate conventional technologies more favorably than genetic technologies, with genetic testing perceived more positively than genome editing. The two additional derived versions, GTQ-H-GR (α = 0.859) and GTQ-MS-GR (α = 0.787), also demonstrated solid psychometric characteristics.

**Conclusion:**

The GTQ-GR is a valid and reliable questionnaire with strong psychometric properties and is now available in Greek.

## Introduction

Rapid developments in genetic technology have made genetic analysis an integral part of clinical practice and research, with genetic testing becoming routine for an increasing number of diseases (Durmaz et al., 2015). Many of the genetic tests are now also available as direct-to-consumer (DTC) tests, i.e., commercially marketed tests that can be used by anyone who wants to pay for them (Shih et al., 2023). These tests may offer the promise of more active self-care and personal autonomy (Flores et al., 2013). However, they often carry more risks than benefits (Lovett and Liang, 2011). Only a limited number of available tests are backed by evidence-based recommendations (Lovett et al., 2012), and there is a lack of provision for genetic counseling, which is particularly crucial, especially in the context of genetic tests.

However, recent years have seen advances not only in genetic testing but also in genome editing, which allows the genetic structure of an organism to be altered by changing, removing, or introducing DNA. Since the discovery of the CRISPR/Cas9 gene scissors by Jinek and colleagues in 2012 (Jinek et al., 2012), gene editing has been frequently used in basic, preclinical, and clinical studies (Zhang et al., 2023). While no genome-editing therapies have yet been approved in Germany, the UK regulator has recently approved the world’s first CRISPR/Cas9 gene-editing therapy to cure sickle cell disease and transfusion-dependent beta-thalassemia (Sheridan, 2023).

From a legal perspective, the European Convention on Human Rights and Biomedicine is currently the only binding instrument under international law that deals specifically with the regulation of human genetic engineering (Vidalis, 2022), “An intervention seeking to modify the human genome may only be undertaken for preventive, diagnostic or therapeutic purposes and only if its aim is not to introduce any modification in the genome of any descendants” (Article 13) (Conseil de l'Europe, 1997). However, only 29 of the 47 member states have ratified the Oviedo Convention. In addition, there are significant differences between national regulations that define how genome editing tools can be used in clinical practice, as well as significant differences in the legal interpretation of the regulatory framework (Nordberg and Antunes, 2022). With regard to germline gene editing for clinical purposes, all gene therapy clinical trials involving germline modifications have been banned by the EU Commission, the European Medicines Agency (EMA), and the Federation of European Academies of Medicine (FEAM) since 2014 (European Union, 2014).

The precautionary principle, which is based on the maxim of “better safe than sorry” (Margolis, 1996), applies not only to interventions in humans but also to the genome modification of plants. In 2018, the European Court of Justice ruled that plant varieties modified by genome editing also fall under Europe’s strict genetic engineering legislation. This should also apply if, in contrast to conventional genetic engineering, no foreign genetic segments are inserted, and the resulting genetic modification is indistinguishable from a conventionally produced modification (Gelinsky and Hilbeck, 2018). Nevertheless, the Director General of the International Food Policy Research Institute warns, “Condemning agricultural biotechnology for its potential risks without considering the alternative risks of prolonging human misery through hunger, malnutrition and the death of children is as unwise and unethical as blindly pursuing this technology without the necessary biosafety” (Caradus, 2023).

The astonishing potential of this new technology for profound intervention in the genomes of humans and other species has triggered a broad debate on the ethical dimension of genome editing, leading to controversy not only among scientists but also among ethicists and policy‐makers (Brokowski and Adli, 2019). In order to engage in this debate, however, we must first examine existing moral views on the value of the human genome, human dignity, social equality and justice, the moral status of unborn and non-consenting human beings, as well as biodiversity, animals, and plants.

The concept of moral status refers to the fundamental position of a being or thing in the normative cosmos. Having moral status means that a being or thing should not be treated arbitrarily on moral grounds but according to certain norms (Schickhardt, 2019). Moral status is granted or withdrawn on the basis of characteristics that are considered morally relevant. While it is generally recognized that humans possess these qualities and should, therefore, be protected for their own sake, it is by no means a foregone conclusion that (all) animals have the qualities considered relevant and can be considered part of the moral community (Grimm et al., 2018). While it is widely accepted that living beings with developed cognitive capacities or who are members of the human species have moral status (DeGrazia, 1996) and should, therefore, be protected for their own sake, preference utilitarians, led by Peter Singer, deny this status to embryos or newborns. They argue that the fetus only receives a weight in the consideration that corresponds to the preference of animals at a similar level (Singer, 2011). Mary Anne Warren also distinguishes between “person” and “biological human”. Cognitive capacities that characterize a person include consciousness, reasoning, self-motivated activity, the ability to communicate, and the presence of self-concepts and self-awareness (Warren, 1973).

The distinction between beings with and without moral status plays a central role in animal ethics and is crucial to the question of the morally correct treatment of animals, i.e., what moral responsibilities we have toward animals (Grimm et al., 2018). Jeremy Bentham was the first to include the pain and suffering of animals in the moral calculus (Bentham et al., 2013). Singer builds on Bentham’s pathocentric criterion of the capacity for suffering or sentience, although the interest in not suffering may conflict with other interests or with the qualitatively equivalent interests of other individuals within the moral community (Singer, 2011). Tom Regan goes beyond Singer’s claim and argues that animals have an inherent value and thus an independent moral status that is associated with entitlements and can, therefore, best be expressed and protected by fundamental, inalienable (moral) rights (Regan, 2019). Most ethicists agree that animals have a higher status than plants (Pouteau, 2014) and – although controversial, as just shown – humans have a higher status than animals and plants.

Although many studies deal with ethical questions in connection with gene editing in humans or embryos or gene editing in the germline (Coller, 2019; Greenfield, 2021), there are only a few studies that deal with the moral evaluation of genetic testing and gene editing in humans as well as in animals and plants, taking into account the moral status of the organisms. As far as we know, the only valid instrument for the moral assessment of genetic technologies was developed by Küchenhoff et al. (2022). Küchenhoff et al. (2022) named the questionnaire the “Genetic Technology Questionnaire” (GTQ) and it was originally developed to assess the moral judgment of laypersons on genetic testing and genome editing and contains six categories: genetic testing of humans and non-humans, genome editing of humans and non-humans, data protection, and social justice. The questions address living beings of different moral status such as embryos, adults, animals, and plants and vary in the severity of the intervention – testing versus editing. As the questionnaire was developed using a contrasting design by replacing items of genetic technology with items regarding conventional technology, e.g., “genetic test” with “ultrasound scan,” the whole questionnaire also exists as “Conventional Technologies Questionnaire” (CTQ) (Küchenhoff et al., 2022).

As views on moral values are linked to cultural norms and values, it is essential to analyze moral judgments on genetic technologies in different countries and cultures. An important aspect of different cultures is religion, which influences morality but is a separate concept. Religions unite people who have similar views on certain things. In religion, justice plays an important role, there are behaviors that apply to everyone, which are indiscriminate. Since religion cannot be debated, it is always and everywhere valid (Thießen, 2014), belief in and affiliation with a particular religion could influence moral views. In order to explore this area of research, valid and reliable instruments are needed. The present study, therefore, aims to translate the Genetic Technology Questionnaire, initially developed in English by Küchenhoff et al. (2022), into Greek and to examine the psychometric properties of the GTQ-GR in its full 30-question format and in the 20- and 5-question short forms, as well as the 5-question version of the CTQ. As the 5-question versions still include the four categories of living beings, but only questions about genome editing and none about genetic testing were raised, we tested additional versions of the GTQ: the GTQ-Human (GTQ-H-GR), which included questions solely about humans and is intended for use in human research and therapy, and the GTQ-Moral Status (GTQ-MS-GR), which contained questions about gene testing and gene editing of different living beings, in order to investigate differences in moral status.

Given that Küchenhoff et al. (2022) have explored various hypotheses regarding the correlation between moral judgments of genetic technologies and socio-demographic variables, and considering our previous examination in a German sample (Teichmann et al., 2025), we intend to extend this comparison to our Greek sample and investigate Küchenhoff’s hypotheses as well. Additionally, we tested the following hypotheses to obtain data for the validation: Conventional techniques will be judged morally better than genetic technologies. Genetic testing will be judged morally better than genome editing. Human genome editing is morally worse than non-human genome editing. Genome editing of embryos is morally worse than that of adults. Religious people judge genetic technologies to be morally worse than non-religious people. Men achieve a higher mean GTQ total score than women. Moral judgment depends on knowledge of genetics.

## Materials and methods

### Study design

This is a cross-sectional study to evaluate the psychometric properties of the Greek version of the GTQ (GTQ-GR). The study followed the EQUATOR guidelines for reporting research using the “Strengthening the Reporting of Observational Studies in Epidemiology” (STROBE) checklist (Elm et al., 2007) (Supplementary Material S1).

### Participants

Between January and September 2023, a convenience sample was recruited through flyers and newsletters and by forwarding the call to participate in the study via social media such as WhatsApp, Viber, and Facebook. Inclusion criteria were age over 18 years and a high Greek language proficiency. To ensure sufficient statistical power in our analyses, we conducted a power analysis using GPower 3.1.9.7 software (Faul et al., 2007), following Kang (2021). The expected effect size for the hypotheses was set at a minimum of d = 0.5, with a desired power of 1 - β = 0.95 and a significance level of α = 0.05. Taking into account our previous studies (Teichmann et al., 2022; Melchior and Teichmann, 2023), which indicated a slightly over-educated sample due to our recruitment approach, we set the allocation ratio to 3. This adjustment anticipated a higher percentage of individuals with advanced knowledge of genetic technologies in the sample, necessitating a larger sample size. GPower recommended a total sample size of N = 244 for the Wilcoxon-Mann-Whitney tests to meet the desired power level. Memon et al. (2020) and Polit (2008) recommend 5 to 20 participants per item for questionnaire-based studies, suggesting a required sample size of 150–300 participants.

The final sample was N = 250, with some participants completing the questionnaire a second time after 4 weeks, resulting in a subsample of n = 50.

### Questionnaire design

The research team used Google Forms to collect data. Participants completed a sequential response process that included several categories, starting with three questions on religiosity, followed by questions about previous experiences with genetic testing. Participants also self-assessed their knowledge about genetic technologies in comparison to the general population. The survey contained 16 questions on knowledge of modern genetics and genomics [adapted from Carver et al. (2017)] and 12 questions on awareness of genetic testing [adapted from (Henneman et al., 2013; Chokoshvili et al., 2017; Chin and Tham, 2020)], from which we only used “I would take a test to create a genetic profile to find out if I am at risk of developing certain diseases”. Participants then completed the 30 questions of the Greek Genetic Testing Questionnaire (GTQ-GR) and five questions of the Greek Conventional Technologies Questionnaire (CTQ5-GR) (Küchenhoff et al., 2022), which is the shortened form of the CTQ.

At the end of the questionnaire, participants were given the opportunity to indicate their interest in participating for a second time after an interval of 4 weeks. They were also given the option to create a code to facilitate the matching of data from both surveys. In addition, participants were requested to provide their email addresses in order to be reminded to participate in the second round. The estimated time required to complete the entire questionnaire was between 10 and 20 min.

### Knowledge about Modern Genetics and Genomics (KMGG)

The KMGG is a component of the “Public Understanding and Attitudes towards Genetics and Genomics” questionnaire, developed by Carver et al. (2017). Designed to assess an individual’s objective knowledge in the field of genetics and genomics, the KMGG focuses on three key areas: (1) characteristics of the genome, (2) gene function and expression, and (3) epigenetics. It consists of 16 statements that respondents answer as “true,” “false,” or “I don’t know.” The total score ranges from 0 to 16 points, with higher scores indicating a more comprehensive understanding of genetics and genomics.

In the original study by Carver et al. (2017), the questionnaire demonstrated reliability, with Cronbach’s alpha coefficients of 0.69 and 0.70. In particular, the Greek and German versions of the questionnaire on knowledge of modern genetics and genomics were validated in a parallel project led by Melchior et al. (2024) and achieved overall very robust properties with Cronbach’s alpha values of 0.85 and 0.84.

### Genetic Technologies Questionnaire (GTQ) and Conventional Technologies Questionnaire (CTQ)

The GTQ, developed by Küchenhoff et al. (2022), is designed to assess lay moral judgments about genetic testing and genetic editing in different living beings, as well as privacy concerns and social justice issues. To examine differences in moral status, both human and non-human species were considered, while the severity of the intervention was contrasted by including items related to genetic testing and editing. The questionnaire encompassed six domains: human genetic testing, non-human genetic testing, human genome editing, non-human genome editing, privacy, and social justice. To prevent framing effects, both the items and response options were neutrally worded.

The GTQ is available in different versions, including the 20-item questionnaire (GTQ20), which contains items that have the highest overall correlations with the total score, and the 5-item questionnaire (GTQ5), which specifically addresses the topics of human and non-human genome editing. Küchenhoff et al. (2022) reported Cronbach’s alpha values of 0.95 for the 30-item version and the 20-item version, and 0.89 for the 5-item version (Küchenhoff et al., 2022).

Using a contrastive design (Burstin et al., 1980), an almost identical item was developed for each GTQ item, with the genetic technologies term replaced by a conventional technologies term (e.g., vaccine instead of genome editing), to form the Conventional Technologies Questionnaire (CTQ). In the present study, only the CTQ5 was used, and ratings of moral goodness or badness were provided on a 6-point Likert scale, ranging from (1) “morally bad” to (6) “morally good” for all scales. Küchenhoff et al. (2022) did not report the validation indices for the CTQ in their publication.

### Developing the Greek version of the questionnaires

The translation-back method (Hambleton, 2001) was used to translate the English version of the GTQ into Greek. Specifically, two native Greek speakers translated the original English version into Greek. Differences in translation were discussed with the research team, ensuring cultural adaptation, and a synthesis of the two translations was produced. This Greek version was back-translated by a bilingual translator. The original English version and the back-translated versions were compared for consistency, relevance and meaning of the content. The Greek version was reviewed by five researchers with expertise in either genetics or ethics to ensure that all items were consistent before finalizing the Greek version of the questionnaires (GTQ-GR, CTQ-GR). The Greek version can be found in Supplementary Material S2.

### Statistical analysis

The analysis of the data involved the application of both descriptive and inferential statistical methods, utilizing IBM SPSS Statistics Version 29 (IBM Corp, 2022). An assessment of the psychometric properties was conducted for all questionnaires that measured a psychological construct. This evaluation included internal consistency (Cronbach’s alpha), structural validity (utilizing principal component analysis), construct validity (employing the known-groups method), item analysis, examination of floor and ceiling effects, and assessment of retest reliability. Additionally, a heatmap provides an overview of the correlations among all relevant variables.

There was no missing data, as Google Forms only accepted completed records.

### Internal consistency and retest reliability

For assessing internal consistency, Cronbach’s alpha was computed to gauge the degree of shared variance among items and to evaluate the reliability of scales comprising more than 10 items. The recommended range for Cronbach’s alpha is 0.70–0.90 (Tavakol and Dennick, 2011).

To evaluate retest reliability, we compared the data from the full sample (N = 250) with those from a subsample (n = 50) after a 4-week interval. The intraclass correlation coefficient was calculated to measure the similarity between the two surveys. Following the method outlined by Koo and Li (2016), the retest-reliability was computed in SPSS using a two-way mixed effects model with the mean of k measurements and absolute agreement.

### Structural validity

A principal components analysis (PCA) with varimax rotation was performed on the GTQ-GR to examine the factor structure. The criteria for conducting PCA included a Kaiser-Meyer-Olkin coefficient (KMO) greater than 0.6 and a significant Bartlett’s test of sphericity with p < 0.05 (Kaiser and Rice, 1974; Tabachnick and Fidell, 2014) and an anti-image correlation, which is a measure of sampling adequacy, of > 0.6 for every item (Grimm et al., 2018). In the PCA, the Kaiser criterion is used to determine the number of factors, extracting the factors that have an eigenvalue greater than 1 (Tabachnick and Fidell, 2014).

### Construct validity

We assessed construct validity using the known-groups method, which distinguishes two distinct groups based on expected differences in their scale scores. Separate known-groups analyses were conducted for the GTQ-GR and CTQ5-GR, utilizing age, gender, religiosity, and education as grouping variables. We formulated the following hypotheses for this study: (1) Older individuals (50+ years) score lower on the GTQ-GR compared to younger individuals (18–30 years). (2) Women score lower on the GTQ-GR than men. (3) Individuals with high religiosity score lower on the GTQ-GR than those with low religiosity. Religiosity was assessed by participants’ frequency of attending church services, their self-perceived level of religiousness, and the extent to which their opinions are influenced by religion, with a scale ranging from 3–15 (3-5 considered less religious, 11–15 considered more religious). (4) Individuals with higher levels of education obtain higher scores on the GTQ-GR compared to those with lower education levels. Education levels were categorized as “lower education” (no academic qualification) and “higher education” (a bachelor’s degree or higher). These hypotheses were tested using either Wilcoxon-Mann-Whitney tests (WMW) (Mann and Whitney, 1947) or Kruskal-Wallis tests (Kruskal and Wallis, 1952).

### Item analysis

We conducted an item analysis for each questionnaire to assess the item-total correlation for all items. This correlation measures the consistency between the score of an individual item and the total scale score and provides valuable insight into the explanatory power of each item. In addition, we examined inter-item correlations to assess the strength of relationships between items.

Typically, mean item-total and mean inter-item correlations in the range of 0.2–0.4 are considered indicative of a significant contribution of information to the scale. However, it is important to note that higher correlations do not necessarily imply higher reliability. Excessively high correlations may indicate item redundancy, leading to an artificial inflation of reliability (Ferketich, 1991; Rattray and Jones, 2007; Piedmont, 2014).

### Floor and ceiling effects

Ceiling or floor effects occur when observations reach the maximum or minimum values, such as a perfect score. When a variable clusters at these extreme values, it introduces bias and distorts the distribution, potentially causing misleading results in analyses that assume a normal distribution (Šimkovic and Träuble, 2019). Although specific thresholds for this effect are not universally defined and range from no specific threshold to a 20% cutoff value (Cramer and Howitt, 2006; Springer, 2014), we considered it to be present if more than 10% of all participants scored at the minimum or maximum level on any questionnaire. In the past, we have successfully demonstrated when a questionnaire exhibited clustering at the ends of the scales and found 10% to be a sufficiently large threshold to indicate a significant problem with the distribution (Melchior and Teichmann, 2023; Melchior et al., 2024).

Furthermore, we reassessed floor and ceiling effects, particularly for groups with low and high self-rated knowledge of genetic technologies, to determine whether the presence of a floor or ceiling effect depended on the specific sample.

### Pairwise comparisons of the GTQ domains

We conducted a Friedman two-way analysis of variance with ranks, followed by a Dunn-Bonferroni post-hoc test, to examine pairwise differences between the eight different domains of the GTQ-GR: (1) genetic testing on humans, (2) embryos, (3) plants, and (4) animals, as well as (5) genetic modification on humans, (6) embryos, (7) plants, and (8) animals (Friedman, 1937; Dinno, 2015). The Friedman test, a non-parametric ANOVA, assigned ranks to the participants’ ratings for each of the eight categories and determined whether significant differences existed between these categories. The Dunn-Bonferroni test then identified pairs with significant differences and quantified the magnitude of these differences using a z-statistic.

### Ethical considerations

This study was approved by the Scientific and Ethics Committee of the Greek Association of Alzheimer’s Disease and Related Disorders (GAADRD, Approved Meeting Number: 084/18-01-2023). All procedures involved in this work conformed to the ethical standards of the Declaration of Helsinki, as applicable to national and institutional human experimentation committees. Prior to the survey, the research team obtained written informed consent from each participant. The participants were informed that the research was voluntary, confidential, and for academic purposes only. After merging the data from different time points to ensure anonymity, the research team removed the email addresses from the server.

## Results

### Participants

A total of 250 people participated in the study, 50 of whom completed the questionnaire a second time after 4 weeks. Table 1 shows the sociodemographic data for both the total sample and the subgroup. The average age of the total sample is 41.20 years, with the majority of participants being female (64.4%). The most common level of education attained is a Master’s degree or diploma, accounting for 42.0% of the total sample. The participants were primarily employed in academic positions (20.4%) and other professions (32.0%).

**TABLE 1:** Participant characteristics of the total sample and the subgroup.

*Characteristics*	Full sample (N = 250)		Subgroup^a^ (n = 50)	
	*n*	*%*	*n*	*%*
Age				
Mean (SD)	41.20 (13.16)		39.12 (13.53)	
Gender				
Male	89	35.6	20	40.0
Female	161	64.4	30	60.0
Diverse	0	0.0	0	0.0
Education				
9 years or less	5	2.0		
10 years	1	0.4		
12–13 years	17	6.8		
Vocational training	32	12.8		
Bachelor	56	22.4		
Master/Diploma	105	42.0		
PhD	34	13.6		
Others	0	0.0		
Occupation				
School student	0	0.0		
Student	22	8.8		
Unemployed	17	6.8		
Retiree	20	8.0		
Care profession	16	6.4		
Therapeutical profession	29	11.6		
Physician	15	6.0		
Academic	51	20.4		
Others	80	32.0		
Marital status				
Divorced	16	6.4	5	10.0
In partnership	55	22.0	15	30.0
Single	65	26.0	14	28.0
Married	113	45.2	16	32.0
Widowed or deceased partner	1	0.4	0	0.0
Do you have children?				
yes	113	45.2	22	44.0
no	137	54.8	28	56.0
Have you ever had a genetic test done?				
yes	26	10.4	6	12.0
no	224	89.6	44	88.0
Has genetic testing ever been performed on a close friend or relative?				
yes	60	24.0	17	34.0
no	83	33.2	14	28.0
I don’t know	107	42.8	19	38.0
Would you like to have a genetic test performed?				
yes	66	26.4	22	44.0
no	107	42.8	11	22.0
I don’t know	77	30.8	17	34.0

^a^ Subgroup after 4 weeks for the retest.

When asked about genetic testing, 10.4% of the total sample reported having undergone genetic testing. An additional 24.0% of respondents had a close family member who was genetically tested. While 26.4% of the total sample expressed a desire to undergo genetic testing, 42.8% were not interested in genetic testing.

Religiosity was assessed by three questions about self-rated religiosity, frequency of attending religious services, and how religion influences decisions, which were summed. The mean score was M = 7.41 (SD = 3.137) on a scale of 3–15. We divided the variable into high (11–15), medium (6–10), and low (3–5) and found that most individuals (n = 133) have a medium level of religiosity, while 75 individuals have a low level of religiosity, and only 42 people report a high level of religiosity.

In assessing participants’ self-perceived knowledge of gene technologies compared to the general population, the mean value was M = 3.66 (SD = 1.631) on a 1 to 7 scale. Notably, the majority fell into the “medium” (3–5 points, n = 107) and “high” (6–7 points, n = 77) categories, while 66 individuals rated their knowledge as low (1–2 points). When asked about specific genes for which they had been tested, respondents frequently mentioned those associated with breast cancer, Down syndrome (trisomy 21), Alzheimer’s disease, and general cancer susceptibility.

Participants who expressed a desire to undergo genetic testing in the future were asked about their motivations. The most commonly cited reasons included concerns about cancer, dementia, Parkinson’s disease, family history, fear of disease, prevention, and early detection. In addition, some participants expressed an interest in testing for all possible conditions or out of curiosity without a specific reason.

### Genetic Technologies Questionnaire (GTQ)

#### Correlations

Table 2 provides an overview of the correlations between the GTQ-GR, other GTQ-GR variants, and the variables considered in this dataset. All presented correlations were analyzed using Spearman’s rank correlation.

**TABLE 2:** Heatmap for the correlations between the questionnaires and other variables.

Scale/Item	2	3	4	5	6	7	8	9	10	11	12	13	14	15
1. GTQ-GR	**0.97**	**0.89**	**0.80**	**0.92**	**0.89**	**0.60**	0.00	−0.03	**−0.14**	0.02	0.01	0.14	**0.19**	−0.06
2. GTQ20-GR	1.00	**0.93**	**0.83**	**0.85**	**0.90**	**0.52**	−0.03	0.00	**−0.15**	0.05	0.05	0.11	**0.16**	−0.09
3. GTQ5-GR	**0.93**	1.00	**0.84**	**0.78**	**0.78**	**0.41**	−0.07	−0.01	**−0.14**	0.00	0.04	0.13	0.13	−0.06
4. CTQ5-GR	**0.83**	**0.84**	1.00	**0.65**	**0.75**	**0.44**	−0.06	0.07	−0.12	0.10	**0.14**	0.14	0.09	0.01
5. GTQ-H-GR	**0.85**	**0.78**	**0.65**	1.00	**0.75**	**0.66**	0.00	−0.09	**−0.17**	−0.05	−0.10	**0.17**	**0.22**	−0.05
6. GTQ-MS-GR	**0.90**	**0.78**	**0.75**	**0.75**	1.00	**0.61**	0.02	0.04	**−0.17**	0.07	0.08	0.12	**0.16**	−0.05
7. Human Testing^a^	**0.52**	**0.41**	**0.44**	**0.66**	**0.61**	1.00	0.03	−0.07	**−0.18**	0.03	0.04	**0.22**	**0.15**	0.06
8. Age	−0.03	−0.07	−0.06	0.00	0.02	0.03	1.00	−0.04	**0.16**	−0.09	0.02	−0.20	−0.07	**−0.18**
9. Years of education	0.00	−0.01	0.07	−0.09	0.04	−0.07	−0.04	1.00	0.03	**0.13**	**0.28**	−0.08	−0.03	0.07
10. Religion	**−0.15**	**−0.14**	−0.12	**−0.17**	**−0.17**	**−0.18**	**0.16**	0.03	1.00	0.01	**0.15**	−0.09	−0.05	0.01
11. KMGG^b^	0.05	0.00	0.10	−0.05	0.07	0.03	−0.09	**0.13**	0.01	1.00	**0.36**	0.01	−0.13	0.10
12. SAK^c^	0.05	0.04	**0.14**	−0.10	0.08	0.04	0.02	**0.28**	**0.15**	**0.36**	1.00	0.10	−0.06	0.07
13. Genetic test^d^	0.11	0.13	0.14	**0.17**	0.12	**0.22**	**−0.20**	−0.08	−0.09	0.01	0.10	1.00	**0.42**	**0.25**
14. Profiling test^e^	**0.16**	0.13	0.09	**0.22**	**0.16**	**0.15**	−0.07	−0.03	−0.05	−0.13	−0.06	**0.42**	1.00	−0.02
15. Gender^f^	−0.09	−0.06	0.01	−0.05	−0.05	0.06	**−0.18**	0.07	0.01	0.10	0.07	**0.25**	−0.02	1.00

Significant correlations are highlighted in bold.

^a^ Items 1, 2, 3, 4, 21, which include statements about genetic testing for humans.

^b^ Knowledge of Modern Genetics and Genomics questionnaire.

^c^ Self-assessed knowledge about genetic technologies.

^d^ Would you like to have a genetic test performed? (0 = no, 1 = yes, “I don’t know” was excluded).

^e^ I would take a test to create a genetic profile to find out if I am at risk of developing certain diseases (1 = strongly disagree to 5 = strongly agree).

f Male = 0, female = 1; *significant at the level p < 0.05; **significant at the level p < 0.001. The color saturation has been adjusted for better visualization and is not proportional to the numerical value.

#### Descriptive statistics

Table 3 displays the descriptive statistics for the GTQ-GR and all other questionnaires. The GTQ-GR yielded a mean score of 3.852 (SD = 0.828) on a scale ranging from 1 to 6, with all GTQ versions sharing the same range. Supplementary Material S3 additionally contains descriptive statistics for each individual item, as well as item-total correlation, alpha-if-removed, skewness, standardized skewness, kurtosis and standardized kurtosis for all items. The mean score for the GTQ20-GR was comparable to that of the GTQ-GR, with a value of M = 3.690 (SD = 1.000), and the GTQ5-GR had a slightly lower mean of M = 3.503 (SD = 1.250).

**TABLE 3:** Psychometric properties of all tested questionnaires.

Scale	Mean score (SD) [range]	Cronbach’s alpha			Mean item-total correlation	Mean inter-item correlation
	*Total sample*	*Total sample*	*LK* ^a^	*HK* ^b^	*Total sample*	*Total sample*
GTQ-GR^c^	3.852 (0.828) [1,6]	0.929	0.928	0.928	0.528	0.300
GTQ20-GR	3.690 (1.000) [1,6]	0.935	0.926	0.934	0.624	0.416
GTQ5-GR	3.503 (1.250) [1,6]	0.866	0.858	0.868	0.690	0.568
GTQ-H-GR	3.950 (0.781) [1,6]	0.859	0.867	0.837	0.473	0.263
GTQ-MS-GR	4.259 (0.883) [1,6]	0.787	0.706	0.785	0.492	0.309
CTQ5-GR^d^	3.845 (1.043) [1,6]	0.758	0.723	0.739	0.528	0.382

^a^ LK, low self-assessed knowledge about genetic technologies.

^b^ HK, high self-assessed knowledge about genetic technologies.

^c^ Genetic Technologies Questionnaire.

^d^ Conventional Technologies Questionnaire.

#### Internal consistency and retest reliability

Table 3 provides internal consistency scores for the total sample and is also broken down for the two self-assessed knowledge groups. The Cronbach’s alpha values for GTQ-GR, GTQ20-GR, and GTQ5-GR are excellent, measuring at 0.929, 0.935, and 0.866, respectively. Notably, both GTQ-GR and GTQ20-GR surpass the recommended threshold of 0.9. Even when examining the two groups with high and low self-assessed knowledge of genetic technologies, the internal consistency values remain consistent, showing no sample-dependent weaknesses.

Table 4 presents the results of the retest, showing the intraclass correlation coefficient (ICC) along with its 95% confidence intervals. Notably, all GTQ variants showed acceptable performance during the retest. The GTQ20-GR scored the highest at 0.835, with CTQ5-GR only reaching an ICC of 0.709.

**TABLE 4:** Test-retest reliability with the subgroup (n = 50).

Scale	Intraclass correlation coefficient	95% – Confidence interval	
		*Lower bound*	*Upper bound*
GTQ-GR	0.781	0.600	0.880
GTQ20-GR	0.835	0.696	0.910
GTQ5-GR	0.797	0.628	0.889
GTQ-H-GR	0.757	0.554	0.868
GTQ-MS-GR	0.743	0.528	0.860
CTQ5-GR	0.709	0.448	0.844

#### Structural validity

The criteria for PCA were met with a KMO of 0.901 and a significant Bartlett’s test for sphericity χ^2^ (435) = 4,406.41, p < 0.001. The measure of sampling adequacy (MSA) values, provided in Supplementary Material S4, exceeded 0.6 for all 30 items, with the lowest value being 0.868 for Item 14.

The analysis of the GTQ-GR through PCA revealed the presence of five distinct factors. The first factor, comprised of items 30, 28, 26, 25, 24, 23, 29, and 27 (arranged in descending order of factor loading), focuses on the genetic modification of animals and plants and explained 19.3% of variance. The second factor, encompassing items 19, 18, 17, 21, 22, and 20, addresses aspects related to disease prevention and cognitive enhancement and explained 11.4% of variance. Factor 3 (items 1, 4, 3, 2, 10) is associated with the evaluation of the risk of genetic diseases and explained 11.2% of variance. The fourth factor includes items associated with data protection (13, 11, 14, 9), while the fifth factor revolves around genetic testing in plants and animals, involving items 5, 6, 7, and 8. They explained 10.9% and 8.5% of variance respectively. Notably, three items (12, 15, 16) exhibited ambiguous factor loadings and could not be confidently assigned to any specific factor. The factor loadings and explained variance is included in Supplementary Material S5.

#### Construct validity

Table 5 displays the outcomes of the known-groups method applied to gender, education, age, and religion. The findings indicate that all versions of the GTQ successfully differentiated between participants based on religious affiliation, effectively distinguishing between religious and non-religious individuals. However, none of the variants demonstrated the ability to identify distinctions among genders, age groups, or educational groups.

**TABLE 5:** Known-groups analysis for all questionnaires.

Group (n)	Gender		Education		Age		Religion		*Significance*
	*Female (161)*	*Male (89)*	*Non-academic (56)*	*Academic (194)*	*Younger (67)*	*Older (62)*	*Low (75)*	*High (42)*	
Mean rank									
GTQ-GR	122.44	131.03	134.01	123.04	64.72	65.31	63.97	50.13	Religion*
GTQ20-GR	120.89	133.85	132.67	123.43	65.67	64.27	64.04	50.00	Religion*
GTQ5-GR	122.10	131.65	132.07	123.60	67.70	62.08	64.03	50.00	Religion*
GTQ-H-GR	123.00	130.03	138.79	121.66	64.57	65.46	64.93	48.40	Religion*
GTQ-MS-GR	123.05	129.93	124.41	125.81	63.54	66.57	64.05	48.98	Religion*
CTQ5-GR	126.02	124.57	119.52	127.23	67.29	62.52	63.59	50.80	Religion*

Annotations: WMW, Tests were used for all analyses. **p < 0.001. *p < 0.05.

#### Item analysis

The item analysis of the GTQ-GR indicates overall satisfactory results; however, there are noticeable instances of excessive similarity among certain items. While this suspicion is evident through a qualitative comparison of item content, a quantitative confirmation is obtained by examining inter-item and item-total correlations.

The GTQ-GR exhibits a mean item-total correlation of 0.528, surpassing the recommended threshold of 0.4. Similarly, the GTQ20-GR and GTQ5-GR display even higher mean correlations of 0.624 and 0.690, respectively.

Upon closer inspection of individual items, redundancy is apparent, with some statements contributing little new information due to overlap with other items. For instance, the correlation between item 30 “The genetic modification of plants to improve seed quality is … ” and item 2 “The genetic modification of seeds to improve nutritional value is … ” is notably high at 0.862, rendering them statistically almost identical. Additionally, other items, such as items 8 and 24 to 30, exhibit elevated inter-item correlations as these have similar contents. Despite these concerns, none of the GTQ variants exhibit evidence of floor or ceiling effects.

### Interest in and experience with genetic testing

Furthermore, we investigated the potential impact of 1) whether an individual underwent genetic testing, 2) whether a close friend or relative underwent genetic testing, and 3) whether the participant expressed interest in genetic testing. Using the WMW test, we analyzed whether these groups exhibited differences in their GTQ-GR scores. The corresponding results are presented in Table 6.

**TABLE 6:** Results of the Wilcoxon-Mann-Whitney tests for interest in and experience with genetic technologies.

Item	Mean rank		U^a^	z^b^	p^c^
	*Yes (n = 60)*	*No (n = 83)*			
Has genetic testing ever been performed on a close friend or relative?	79.76	66.39	2024.5	1.905	0.057
	*Yes (n = 109)*	*No (n = 40)*			
Would you like to have a genetic test performed?	78.66	65.03	1781.0	1.709	0.087
	*Yes (n = 26)*	*No (n = 224)*			
Have you ever had a genetic test done?	121.12	126.01	3,026.0	0.327	0.744

^a^ U, U test statistic.

^b^ z, z statistic.

^c^ p, significance, a higher mean rank is associated with a higher mean GTQ-GR score.

No difference in GTQ values was observed among any of the three groups, only minor trends that did not reach statistical significance.

Furthermore, we categorized the participants into three groups: (1) those desiring genetic testing, (2) those not interested in genetic testing, and (3) those uncertain about undergoing genetic testing. In this context, we examined whether this inclination was mirrored in their response to the item “I would take a test to create a genetic profile to find out if I am at risk of developing certain diseases.” The findings are presented in Table 7.

**TABLE 7:** Consistency in the interest for genetic testing.

Item		I Would take a test to create a genetic profile to find out if I am at risk of developing certain diseases				
		*strongly disagree*	*disagree*	*neutral*	*agree*	*strongly agree*
Would you like to have a genetic test done?	yes (n = 109)	1	1	9	47	51
	no (n = 40)	3	5	13	11	8
	I don’t know (n = 101)	2	7	32	41	19

Of the participants who expressed interest in genetic testing, the majority (n = 98 out of 109) confirmed their willingness to undergo genetic profiling. Conversely, among those who denied interest in testing, about half (n = 19 out of 40) expressed interest in genetic predisposition testing. Among those who were undecided, more than half (n = 60 out of 101) expressed a desire to undergo such a test.

#### Moral judgment of the different domains

**FIGURE 1:**
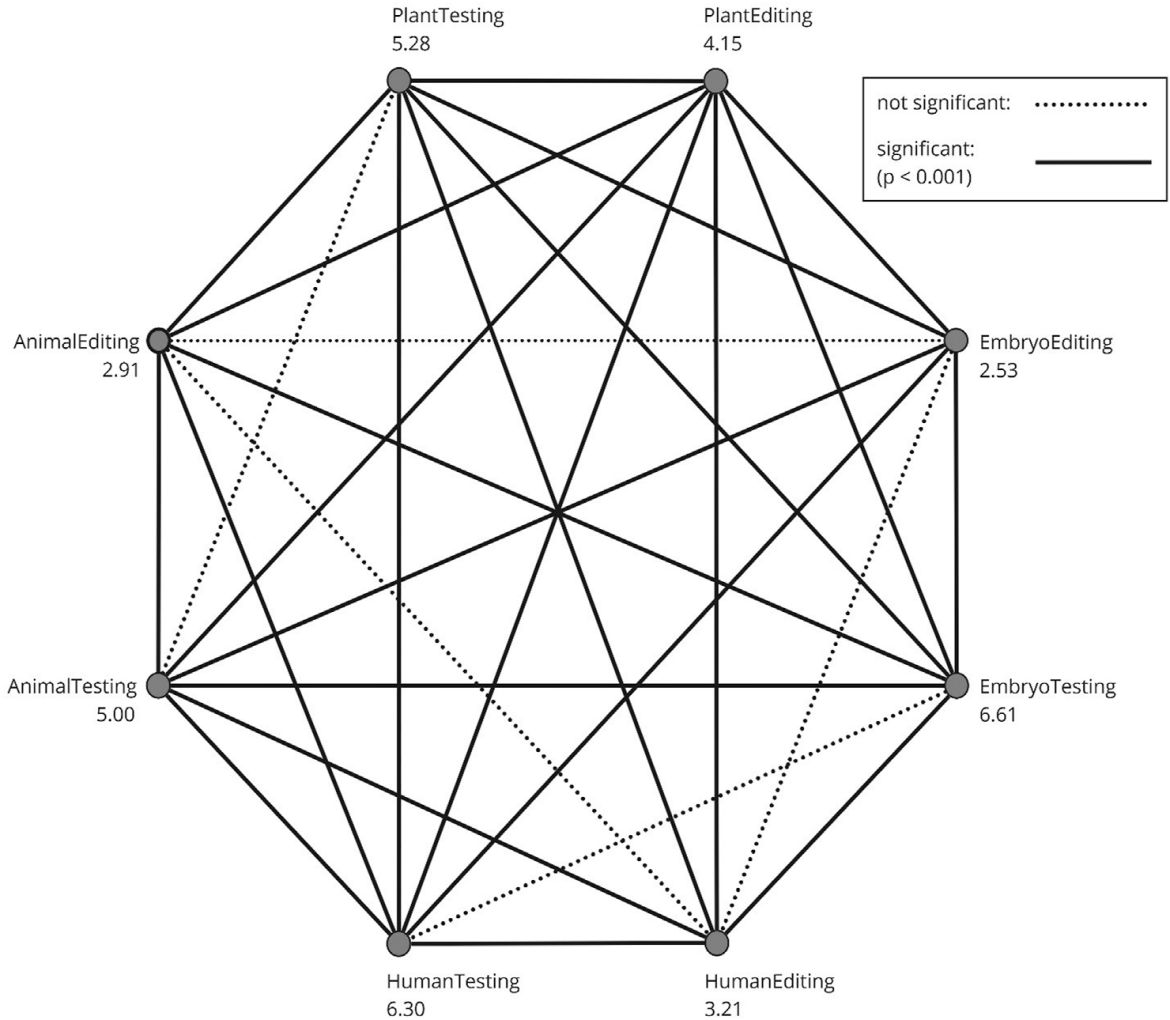
Post-hoc pairwise comparison of the GTQ domains The numbers on the eight categories are the mean ranks from the Friedman test. Pairs with a significant difference relate to a continuous line. A dotted line means that the pair is not significantly different.

To evaluate participants’ perceptions of eight distinct domains—genetic testing on (1) humans, (2) embryos, (3) plants, and (4) animals, as well as genetic modification on (5) humans, (6) embryos, (7) plants, and (8) animals—a Friedman’s two-way analysis of variance by ranks was performed. Subsequently, a Dunn-Bonferroni post-hoc test was employed to identify pairwise differences. Figure 1 illustrates a chart wherein each pairwise comparison was assessed for significance, connecting pairs displaying a significant difference with a continuous line. The numbers assigned to the eight categories represent the average ranks from the Friedman test, ranging from 1 to 8. Several noteworthy findings emerge from this analysis: Genetic testing consistently garners significantly higher moral favorability ratings compared to its genetic editing counterparts, e.g., animal testing and animal editing. The moral rating for genetic modification in embryos registers as the lowest, while genetic testing in embryos receives the highest moral rating. Furthermore, moral ratings for gene editing in animals and adults are comparable and significantly lower than those for genetic modification in plants.

### GTQ-H-GR and GTQ-MS-GR

In addition to the 30-item-GTQ and its various versions, we examined two other categories relevant to the moral assessment of genetic technologies, encompassed by the items of the GTQ-GR. These are the GTQ-Human (GTQ-H-GR), focusing exclusively on human-related items (items 1, 2, 3, 4, 9, 10, 11, 12, 13, 15, 16, 17, 18, 19, 20, 21, and 22), and the GTQ-Moral Status (GTQ-MS-GR), which incorporates items related to moral status (items 1, 2, 6, 7, 17, 20, 29, and 30). The psychometric properties of both questionnaires are detailed in Tables 2–5.

The GTQ-H-GR exhibits very good internal consistency, evident by a Cronbach’s alpha of 0.859, while the GTQ-MS-GR has a slightly lower alpha of 0.787. This discrepancy is anticipated, given that all items cover different factors, and there is no repetition of similar items addressing the same topic within each questionnaire.

The mean item-total correlation for the GTQ-H-GR, at 0.473, is lower than that observed in other GTQ variants, and the GTQ-MS-GR is similar to the GTQ-H-GR, with an item-total correlation of 0.492. The absence of high-correlation items related to plants in these two GTQ variants contributes to an average inter-item correlation of 0.263 for the GTQ-H-GR and 0.309 for the GTQ-MS-GR, representing an optimal value. The test-retest reliability for the GTQ-H-GR was acceptable at 0.757 (95% CI: 0.554; 0.868) and slightly lower for the GTQ-MS-GR at 0.743 (95% CI: 0.528; 0.860).

The PCA of the GTQ-H-GR was not entirely conclusive, and there were a few items that loaded onto all factors. Nevertheless, three factors are discernible, with the first factor consisting of items 19, 18, 17, 21, and 20 (in order of factor loadings) with an internal consistency of 0.790. This factor includes items related to the therapy of genetic diseases through genetic modification methods and the prevention of diseases through gene alteration. The second factor includes items related to data protection and privacy (items 11, 13, 9, and 10) with an internal consistency of 0.716, while the third factor consists of items related to genetic testing (items 4, 2, 3, and 1) and has an internal consistency of 0.785.

In the known-groups analysis, the GTQ-H-GR and GTQ-MS-GR were able to distinguish between religious and non-religious participants but failed to detect any differences concerning gender, age, or education, similar to the other GTQ variants. Neither the GTQ-H-GR nor the GTQ-MS-GR showed evidence of ceiling or floor effects.

### CTQ5-GR

#### Descriptive statistics

The mean score of the CTQ5-GR was 3.85 (SD = 1.043) on a scale of 1–6 and, according to a Wilcoxon signed-rank, the CTQ5-GR had significantly higher scores than the GTQ5-GR (CTQ5-GR: Mdn = 4.0, IQR = 1.25; GTQ5-GR: Mdn = 3.6, IQR = 1.80; z = 7.605, p < 0.001).

In addition, we examined whether religious individuals perceive the statements of the CTQ5-GR as morally better than those of the GTQ5-GR. For this purpose, we used a Wilcoxon signed-rank test to compare the CTQ5-GR and GTQ5-GR scale scores within the same individual. It turns out that a person with high religiosity has a significantly higher total score on the CTQ5-GR compared to the GTQ5-GR (CTQ5-GR Mdn = 3.6, IQR = 1.25; GTQ5-GR Mdn = 2.9, IQR = 1.85; z = 3.889, p < 0.001).

#### Internal consistency and retest reliability

In the total sample, the CTQ5-GR achieved an acceptable Cronbach’s alpha value of 0.758, which was unimpacted by the high knowledge group. With a confidence interval of 0.448–0.844, the retest is somewhat low.

#### Construct validity

Construct validity results closely mirror those of the GTQ versions. The CTQ5-GR discriminated effectively between non-religious and religious individuals but showed no other significant differences across age, gender, and education groups.

#### Item analysis

In the item analysis, the CTQ5-GR demonstrated a mean inter-item correlation of 0.382, which falls within the desired range, and a mean item-total correlation of 0.528, which slightly surpasses it. Notably, item 3 (“Changing the hormones of farm animals to improve their wellbeing is…”) and item 4 (“Changing the hormone balance of farm animals to reduce costs without harming them is…”) exhibit a correlation of 0.63, suggesting substantial redundancy between these two items.

#### Floor and ceiling effects

Approximately 1% of individuals attained the minimum score, while less than 5% achieved the maximum score on the CTQ5-GR. These percentages suggest an absence of distributional bias in the scores.

### Comparison to the original study

Table 8 outlines the hypotheses formulated and tested by Küchenhoff et al. (2022) with a representative sample of the U.S. population. The table shows the results of the original, the German (Teichmann et al., 2025) and the Greek validation study.

**TABLE 8:** Results for hypothesis formulated by Küchenhoff et al. (2022).

No.	Statement	Küchenhoff et al., 2022 (USA)	Teichmann et al., 2025 (Germany)	This sample (Greece)
1	Genetic editing of human adults is regarded as better than that of embryos (the mean rating of GTQ18^a^ is greater than that of GTQ19; that of GTQ15 is greater than that of GTQ22)	**✓**	**✓**	**✓**
2	Overall, ratings of genetic testing (GTQ1-8) correlate with ratings of genome editing (items GTQ15-30)	**✓**	**✓**	**✓**
3	Overall, ratings of genome editing are lower (morally worse) than of genetic testing (the mean rating of GTQ15-30 is lower than that of GTQ1-8)	**✓**	**✓**	**✓**
4	Participants self-identified as male rate the use of genetic technologies on animals (items GTQ5, 6, 23, 24, 27, 29) as morally better than participants self-identified as female	N/A	**✓**	✗
5	Participants rate genetic technologies as morally better when they are used to improve nutritional value (GTQ28) or fight world poverty (GTQ25) than to improve taste (GTQ26)	**✓**	**✓**	**✓**
6	Participants rate genetic technologies as morally better when they are used to improve wellbeing (GTQ23) rather than to increase efficiency (GTQ6)	✗	✗	✗
7	Genome editing of embryos is rated as morally better when performed in order to prevent a fatal disease (GTQ17) than when used to prevent influenza (GTQ19)	**✓**	**✓**	**✓**
8	Genome editing of human adults is rated as morally better when performed in order to treat cancer (GTQ20) than when used to protect them against influenza (GTQ18)	✗	**✓**	✗
9	The higher the participant’s education (measure of education level, years of education) the higher the GTQ total score	**✓**	✗	✗
10	The more religious participants consider themselves to be, the worse they rate genome editing (GTQ15-30)	✗	**✓**	**✓**
11	Participants who already had experience with genetic tests rate genetic technologies as morally better (GTQ total score)	✗	✗	✗
12	The more participants think they know about genetic technologies, the more extreme (trending away from the midpoint of the scale) they rate the morality of genetic technologies (positive or negative)	✗	✗	✗
13	Objective knowledge about genetics is negatively correlated with opposition to genetic technologies	**✓**	**✓**	✗
14	A discrepancy between self-assessed and objective knowledge about genetic technologies is positively correlated with opposition to genetic technologies	✗	✗	✗

^a^ GTQ18 refers to item 18 from the GTQ, so do all the other GTQXs, in this table.

Upon examining the results, notable differences emerge when compared to Küchenhoff et al.’s (2022) sample. Analysis of the first statement reveals that, in line with Wilcoxon signed-rank tests, genetic modification in adults is perceived as significantly more morally acceptable than genetic modification in embryos (GTQ18 and GTQ19: z = 5.720, p < 0.001; GTQ15 and GTQ22: z = 3.922, p < 0.001). Additionally, the correlation between genetic testing (items 1–8) and genetic modification (items 15–22) was confirmed to be significant with ρ = 0.534 (p < 0.001).

Further supporting our findings, the Wilcoxon signed-rank test affirms that genetic testing is morally superior to genetic modification (z = 13.319, p < 0.001). Genetic testing exhibited a mean of 4.904 (SD = 0.823), while genetic modification had a mean of 3.489 (SD = 1.074).

We hypothesized that male participants would perceive the use of genetic technologies on animals as morally superior to female participants. However, this hypothesis was not substantiated in a WMW test (z = 1.320, p = 0.187).

In terms of moral acceptability, improving nutrition (z = 9.404, p < 0.001) and combating poverty (z = 10.783, p > 0.001) were rated higher than enhancing taste. Contrary to hypothesis six, our findings indicate that participants consider the use of genetic technologies for improving efficiency more morally acceptable than for the wellbeing of animals (z = 3.907, p < 0.001).

Confirmation of statement seven was obtained with z = 6.912 and p < 0.001. However, deviating from Küchenhoff et al. (2022), our sample revealed that – for hypothesis 8 – genetic modification for cancer treatment (GTQ20) was morally rated lower than genetic modification for protection against influenza (GTQ18) with z = 3.461, p < 0.001.

Our sample showed no significant correlation between the highest level of education and the GTQ30-GR score (ρ = −0.063, p = 0.318), nor between the number of years of education and the GTQ30-GR score (ρ = −0.034, p = 0.592). However, in this sample, we were able to demonstrate a negative correlation between religiosity and the evaluation of genetic technologies: ρ = −0.142, p = 0.024.

Hypothesis 11 could not be confirmed (z = 0.327, p = 0.744). Moreover, in hypothesis 12, no relationship was found between self-rated knowledge of genetic technologies and the difference from the scale mean of the GTQ30-GR (ρ = 0.043, p = 0.498).

There was no significant correlation between the KMGG score and the GTQ-GR score (ρ = 0.016, p = 0.801). For the final hypothesis, we examined the discrepancy between self-rated knowledge and objective knowledge (KMGG total score) using normalized scale scores. However, the hypothesis could not be confirmed as this discrepancy did not correlate with the GTQ-GR total score (p = 0.203).

## Discussion

### Scale properties

The objective of the present study was to translate the GTQ and its variations into Greek and validate them within the Greek population. All GTQ-GR variants (GTQ-GR, GTQ20-GR, GTQ5-GR, GTQ-H-GR) demonstrated good to excellent internal consistencies, with only the GTQ-MS-GR showing a slightly lower but still acceptable value. The questionnaires showed poor to moderate test-retest reliability, but it is crucial to note that the small number (n = 50) in the sample size of the retest groups could have resulted in a degradation of the reliability assessment (Polit, 2014; Aldridge et al., 2017), leading to underestimation. The PCA revealed the presence of five distinct factors, with three items that could not be assigned to any of the five factors, however, Küchenhoff et al. (2022) did not propose a model structure for the GTQ, which would have been desirable for a psychometric instrument. Having a proposed model would allow for examination using confirmatory factor analysis and enhance the quality of the questionnaire.

Additionally, it should be noted that the GTQ-GR shows a high correlation between items, with no floor or ceiling effects. The GTQ-GR score remained unaffected by factors such as undergoing a genetic test, knowing someone who had been tested, or having a desire to take a genetic test.

In line with the German validation study of the GTQ by Teichmann et al. (2025), our examination indicated that the GTQ-GR is adversely affected by the inclusion of numerous similar items, resulting in unnecessary lengthiness and the supplementary items do not provide any added value. Furthermore, the internal consistency is artificially elevated due to a substantial proportion of shared variance. While the broad factor structure aligns with the factor structure of the German sample (Teichmann et al., 2025), there are notable differences in the known-groups analysis. In the German sample, all GTQ versions could distinctly differentiate between gender, age groups, and religiosity, whereas in this sample we found differences only for religion. The observed phenomenon might stem from a potential bias in the distribution of age and gender, notably with male participants being significantly older than their female counterparts and some groups having very small sizes—such as the fifteen young male participants — leading to reduced testing power. Consequently, we suggest employing a larger sample size for subsequent investigations.

### Religiosity and the impact on the judgement of genetic technologies

In our study, religious individuals perceive genetic technologies as morally worse than less religious participants. This is in accordance with previous studies on the influence of religiosity on genetic testing or genome editing. Genetic technologies may be seen as a mechanism that intervenes in human existence (Kalidasan and Das, 2022). Allum et al. (2014) showed that people with higher levels of religiosity are more reluctant to intervene in the genome of embryos because they believe in the “sanctity of human life” and are more likely to oppose genetic testing on unborn babies. Study participants often expressed their attitudes in terms of religious beliefs, and there was a negative influence of religiosity on the acceptability of genetic testing: those who were more religious were less likely to be tested (Allum et al., 2014). Schwartz et al. (2000) showed that women who described themselves as spiritual were much less likely to be tested for breast cancer than women who were less spiritual if they were at low risk for breast cancer; there was no difference for women who were at high risk. Regarding prenatal genetic testing and abortion among ethnic minorities, Ahmed et al. (2006) reported that religion had a significant impact, but that it was not the main factor influencing their reproductive decisions. Regarding public perceptions of gene therapy, Robillard et al. (2014) found that less religious people would rate genetic technologies as morally better. The research conducted by Küchenhoff and colleagues (2022) did not reveal any significant impact of religiosity on moral judgments.

### Genetic knowledge and the impact on the judgement of genetic technologies

Because public genetic literacy is thought to vary widely and may influence moral judgments about genetic technologies, we asked study participants about their self-perceived knowledge of genetic technologies and we measured their objective knowledge with the KMGG. We hypothesized that the more people think they know about genetic technologies, the better they will evaluate them morally. Neither Küchenhoff et al. (2022) nor our research team could confirm this hypothesis in our earlier study (Teichmann et al., 2025) or in the present study. We did however observe that objective knowledge about genetics exhibits a positive correlation with moral judgements in our previous study (Teichmann et al., 2025), which was confirmed by Küchenhoff et al. (2022) too, but was not present in this sample.

One possible reason is that the Greek group’s knowledge is too limited to significantly impact their moral judgment. An indicator for this is the large difference in self-assessment of the genetic knowledge. While the mean score in the German validation study was 4.31 (Teichmann et al., 2025), the mean score in the Greek study was only 3.66. This reflects the differences in measured objective genetic knowledge that we investigated in another project where we observed a KMGG score of 5.37 for a Greek sample and 8.67 for the German sample (Melchior et al., 2024).

Unfortunately, Küchenhoff et al. (2022) did not provide information on the knowledge level of their sample. However, when we compared the genetic knowledge of the German sample with that of a US sample, we found that both groups were similarly educated (Teichmann et al., 2025). Additionally, the differences in science curricula between the three countries might play a crucial role and must be included in a more thorough investigation.

Nevertheless, the question remains whether knowledge about genetics and genetic technologies is necessary for a moral assessment of these technologies and additional variables should be considered.

### CTQ versus GTQ

Our hypothesis that conventional technologies (CTQ5-GR) are considered morally better than their genetic counterparts (GTQ5-GR) was confirmed, allowing us to replicate the findings of our previous study with the German public. This finding is consistent with the study by Küchenhoff et al. (2022).

However, it must be emphasized once again that we only used the CTQ5 in the present study, which contains questions on all categories of moral status, i.e., embryo, adult, animal, and plant, but no questions on genetic testing or privacy and, therefore, has a lower mean score than all other versions of the GTQ.

### GTQ-MS-GR

Because the GTQ was designed to take into account the moral status of different living beings, we developed a version of the questionnaire that covers the four different groups of living beings – each with respect to genetic testing and genome editing – the GTQ-MS-GR. We replicated most of the results of our previous study in the German public: Genetic testing was rated as morally better than genome editing, regardless of the species studied. Also, genetic editing in plants was seen as morally superior to editing in animals, as was genetic testing in plants in contrast to animals. However, in the German public, genetic editing in embryos was rated morally worse than editing in human adults, which was not the case in the Greek sample. Küchenhoff and colleagues (2022) have already shown that moral judgments depend on both moral status and the severity of the reason for an intervention. As they did not further analyze their findings with respect to moral status, no further comparisons can be reported. The 8-item questionnaire demonstrated strong internal consistency, showing no excessive correlations between items. Its characteristics are nearly identical to the German version (Teichmann et al., 2025), except for the retest reliability, which was significantly higher in the German sample and should be investigated further in future studies. The questionnaire is, therefore, suitable for studies on the moral status of different living beings or studies on model organisms. The GTQ-MS-GR also appears to be useful for investigating laypersons’ perceptions of animal experimentation.

### GTQ-H-GR

For ethics studies involving only humans, we thought it would be beneficial to test a version of the questionnaire containing only questions about genetic testing and editing and human privacy – the GTQ-H-GR – and to examine the psychometric properties. As reported earlier, the 17-item questionnaire has an excellent internal consistency, with optimal inter-item correlations and an acceptable test-retest reliability. In addition, a three-factor structure was identified for the scale, which divides the GTQ-H-GR into three thematic areas: genetic modification, genetic testing, and privacy, as already shown for the German version of the questionnaire (Teichmann et al., 2025).

In comparison to the structure of the GTQ-H in the German sample, few differences are discernible. Some of the items do not have the exact same factor loadings, which is to be expected, given that PCA is highly dependent on the sample. The internal consistencies of the factors for testing and genetic modification are slightly lower than in the German version; however, the consistency for privacy is higher.

Apart from the PCA, the GTQ-H-GR achieved excellent values in this sample, as it did in the German sample.

We believe that this scale is an appropriate tool for studying the moral evaluation of gene therapy and genetic testing in both embryos and adults in the future. Due to the availability of genetic tests for more and more diseases and in view of the approval of the first gene-editing therapy with the Crisp-Cas9 gene scissors (Sheridan, 2023), it is important to examine the moral evaluation of the public in order to lead the discourse not only among scientists, ethicists, and politicians but also to involve citizens in the discussion.

### Limitations

While we endeavored to ensure a diverse sample by including individuals from various social groups and encourage their social circles to participate in the study, a comparison of our sociodemographic data with the general population in Greece reveals a potential higher level of education in our sample (Statista, 2022). This bias is a recognized challenge, likely stemming from the lower participation of less educated individuals in scientific projects. Furthermore, the recruitment channels we employed predominantly targeted individuals with a specific interest in research projects.

The limitations associated with the online survey format need careful consideration. Online surveys tend to attract respondents who are technologically proficient or have ample free time (Wright, 2005; Hunter, 2012), introducing a potential selection bias. Additionally, as observed in face-to-face interviews, the absence of personal interaction restricts the opportunity to explore more detailed or nuanced responses (Ball, 2019). Technical difficulties, such as slow loading times or issues with the survey software, can frustrate participants and potentially impact response rates.

For future projects, we recommend employing larger sample sizes and implementing an expanded recruitment program. Convenience samples, in general, tend to exhibit statistical bias due to their composition being predominantly WEIRD (Western, Educated, Industrialized, Rich, and Democratic) (Henrich et al., 2010), limiting the ability to generalize and make cross-cultural comparisons.

Although the statistical power was deemed sufficient for the total sample, certain groups in the known-groups analysis were too small to be considered adequate for the hypothesized effect. Consequently, future projects should involve additional participants.

Floor and ceiling effects are not strictly defined in statistics (Cramer and Howitt, 2006), so our definition, while carefully selected based on prior experience, is not universally applicable. It's crucial to note that these effects are highly dependent on the sample and are meant to give only a rough impression of the score distribution to identify potential further issues.

An inherent issue in this study is the potential for respondent fatigue resulting from completing many consecutive scales. The order of these scales was not randomized due to limitations in our survey instrument.

## Conclusion

In summary, the Greek versions of the GTQ questionnaires (GTQ-GR, GTQ20-GR, GTQ5-GR, GTQ-H-GR and GTQ-MS-GR), along with the Greek CTQ5 questionnaire, demonstrate promising characteristics and exhibit no significant shortcomings. Depending on economic considerations, it is advisable to assess whether the utilization of the lengthy 30-item GTQ questionnaire is necessary. However, we discourage the use of the brief 5-item versions due to their limited substance and vulnerability to item redundancy. The derived GTQ-MS-GR and GTQ-H-GR, strengthened by the removal of similar items, are psychometrically more robust and are excellent candidates for future studies.

## Data Availability

The datasets presented in this study can be found in online repositories. The names of the repository/repositories and accession number(s) can be found below: OSF (Genetic Technologies Questionnaire - Greek Validation: https://osf.io/yud83/).

## Abbreviations

CTQ, Conventional Technologies Questionnaire; DNA, deoxyribonucleic acid; GR, Greek; GTQ, Genetic Technologies Questionnaire; GTQ-H, GTQ-Human; GTQ-MS, GTQ-Moral Status; ICC, Intraclass Correlation Coefficient; KMGG, Knowledge about Modern Genetics and Genomics; KMO, Kaiser-Meyer-Olkin coefficient; MSA, Measure of Sampling Adequacy; PCA, principal component analysis; STROBE, Strengthening the Reporting of Observational Studies in Epidemiology; WEIRD, Western, Educated, Industrialized, Rich, and Democratic; WMW, Wilcoxon-Mann-Whitney test.
