# Supplementary material for: The genetic technologies questionnaire in the Greek-speaking population: the moral judgement of the lay public
Source: Front Genet. 2025 May 13;16:1594724. doi: 10.3389/fgene.2025.1594724 (PMC12106406; doi:10.3389/fgene.2025.1594724)
Supplement: Supplementary file 1 [file DataSheet2.pdf]

„Genetic Technologies Questionnaire“ (Küchenhoff et al. 2022) – Greek Translation

	English (Küchenhoff et al. 2022)	Ελληνικά
1	Genetic testing to determine the risk of Down’s syndrome for an embryo in utero is…	Ο γενετικός έλεγχος για να καθοριστεί ο κίνδυνος εμφάνισης συνδρόμου Down για ένα έμβρυο στη μήτρα είναι ...
2	Prescribing genetic tests for healthy women in order to identify markers for breast cancer is …	Η συνταγογράφηση γενετικών εξετάσεων σε υγιείς γυναίκες με σκοπό τον εντοπισμό δεικτών καρκίνου του μαστού είναι …
3	Using genetic tests to determine if one carries markers for hereditary diseases before deciding to conceive a child is …	Η χρήση γενετικών εξετάσεων για να προσδιοριστεί εάν κάποιος φέρει δείκτες για κληρονομικές ασθένειες πριν την απόφαση σύλληψης παιδιού είνα ...
4	Performing genetic tests on consenting adult humans for medical research is …	Η διενέργεια γενετικών εξετάσεων σε συναινούντες ενήλικες ανθρώπους για ιατρική έρευνα είναι ...
5	Conducting harmless genetic tests on animals for scientific research is…	Η διενέργεια ακίνδυνων γενετικών δοκιμών σε ζώα για επιστημονική έρευνα είναι …
6	*Optimizing the breeding of farm animals through genetic testing is…*	*Η βελτιστοποίηση της εκτροφής ζώων φάρμας μέσω γενετικού ελέγχου είναι ...*
7	*Performing invasive genetic tests on wild plants to monitor and conserve ecosystems is…*	*Η διενέργεια επεμβατικών γενετικών δοκιμών σε άγρια φυτά για παρακολούθηση και διατήρηση των οικοσυστημάτων είναι ...*
8	*Genetic testing of crops to improve them for farming is…*	*Ο γενετικός έλεγχος των σοδειών για τη βελτίωσή τους στην καλλιέργεια είναι …*
9	Consider a patient with a hereditary disease who has a sibling with similar genes. For the doctor, informing the sibling of the patient’s disease despite privacy concerns is…	Σκεφτείτε έναν ασθενή με κληρονομική ασθένεια που έχει έναν αδερφό/ή μεπαρόμοια γονίδια. Για τον γιατρό, το να ενημερώσει τον αδερφό/ή του ασθενούς για την ασθένεια, παρά το ιατρικό απόρρητο είναι ...
10	Supporting genetic testing despite privacy concerns is…	Η υποστήριξη του γενετικού ελέγχου παρά τις ανησυχίες για το απόρρητο είναι …
11	For insurers, requesting genetic tests from healthy adults in order to assess their health risks is …	Η απαίτηση γενετικού ελέγχου υγιών ενηλίκων από τις εταιρίες ασφάλισης, με σκοπό την εκτίμηση των κινδύνων υγείας είναι ...
12	*Using public health funds on expensive gene therapies is …*	*Η χρήση κονδυλίων δημόσιας υγείας σε ακριβές γονιδιακές θεραπείες είναι …*
13	Taking into account the genetic profile of applicants with respect to genetic diseases when hiring a kindergarten teacher is…	Η συνεκτίμηση του γενετικού προφιλ των αιτούντων όσον αφορά τις γενετικές ασθένειες κατά την πρόσληψη νηπιαγωγών είναι ...
14	Mitigating a criminal sentence due to the offender’s genetic predisposition is …	Η ελάφρυνση της ποινής λόγω γενετικής προδιάθεσης του δράστη είναι ...

15	*Using genome editing on consenting adults to enhance their cognitive performance is …*	*Η χρήση της επεξεργασίας του γονιδιώματος σε συναινούντες ενήλικες με σκοπό την ενίσχυση της γνωστικής τους επίδοσης είναι ...*
16	*Changing the genomes of human embryos for medical research without destroying them is…*	*Η αλλαγή του γονιδιώματος των ανθρώπινων εμβρύων για ιατρική έρευνα χωρίς την μετέπειτα καταστροφή τους είναι …*
17	*Changing the genomes of human embryos to ensure they will not develop a fatal disease is …*	*Η αλλαγή γονιδιώματος ανθρώπινων εμβρύων για να διασφαλιστεί ότι δεν θα αναπτύξουν μια θανατηφόρα ασθένεια είναι …*
18	***Genome editing of human adults to protect them against influenza is …***	***Η επεξεργασία του γονιδιώματος ενηλίκων για την προστασία τους από τη γρίπη είναι ...***
19	***Changing the genome of human embryos to ensure they will not get influenza is …***	***Η αλλαγή γονιδιώματος ανθρώπινων εμβρύων για να διασφαλιστεί ότι δεν θα αποκτήσουν γρίπη είναι …***
20	*Using risky genome editing therapies for the medical treatment of cancer patients is …*	*Η χρήση επικίνδυνων θεραπειών επεξεργασίας γονιδιώματος για τη θεραπεία ασθενών με καρκίνο είναι ...*
21	*Testing for the risk of genome editing on consenting adults is …*	*Ο έλεγχος του κινδύνου της επεξεργασίας γονιδιώματος σε συναινούντες ενήλικες είναι ...*
22	*Using genome editing to enhance the cognitive development of human embryos in underprivileged families is …*	*Η χρήση επεξεργασίας γονιδιώματος για την ενίσχυση της γνωστικής ανάπτυξης ανθρώπινων εμβρύων μη προνομιούχων οικογενειών είναι …*
23	***Changing the genome of farm animals in order to improve their wellbeing is …***	***Η αλλαγή γονιδιώματος των ζώων εκτροφής (φάρμας) προκειμένου να βελτιωθεί η ευημερία τους είναι ...***
24	***Editing the genome of farm animals to reduce costs without harming them is …***	***Η επεξεργασία γονιδιώματος ζώων εκτροφής (φάρμας) για τη μείωση κόστους χωρίς πρόκληση βλάβης είναι ...***
25	*Editing the genome of crops in order to fight world poverty is …*	*Η επεξεργασία γονιδιώματος των σοδειών για την καταπολέμηση της παγκόσμιας φτώχειας είναι ...*
26	*Editing the genome of foods to improve their taste is …*	*Η επεξεργασία γονιδιώματος σε τρόφιμα με σκοπό τη βελτίωση της γεύσης είναι ...*
27	*Editing the genome of animals to make it possible for animal organs to be transplanted to humans is …*	*Η επεξεργασία γονιδιώματος σε ζώα για να καταστεί δυνατή η μεταμόσχευση των οργάνων τους σε ανθρώπους είναι ...*
28	Editing the genome of crops to improve their nutritional value is…	Η επεξεργασία του γονιδιώματος των σοδειών με σκοπό τη βελτίωση της διατροφικής τους αξίας είναι ...
29	*Editing the genome of wild animals to make them immune against certain diseases is …*	*Η επεξεργασία γονιδιώματος σε άγρια ζώα με σκοπό την ανοσία τους σε συγκεκριμένες ασθένειες είναι ...*

30	***Editing the genome of plants to improve crops for farming is…***	***Η επεξεργασία γονιδιώματος σε φυτά με σκοπό τη βελτίωση της καλλιέργειάς τους είναι ...***
Answers were given on a 6-point Likert scale ranging from (1) “morally bad” to (6) “morally good”. Italicized items are included in the GTQ20, bold items are included in the GTQ5.		Οι απαντήσεις δόθηκαν σε 6βάθμια κλίμακα Likert, από (1) "ηθικά κακό" έως (6) "ηθικά καλό". Τα αντικείμενα με πλάγιους χαρακτήρες περιλαμβάνονται στο GTQ20, τα αντικείμενα με έντονους χαρακτήρες περιλαμβάνονται στο GTQ5.

Conventional Technologies Questionnaire 5 (CTQ5) – Greek Translation

	English (Küchenhoff et al. 2022)	Greek
1	Vaccinating human adults to protect them against influenza is …	Ο εμβολιασμός των ενηλίκων για την προστασία από τη γρίπη είναι ...
2	Using vaccination on human embryos to ensure they will not get influenza is …	Η χρήση εμβολιασμού σε ανθρώπινα έμβρυα για την προστασία από τη γρίπη είναι ...
3	Changing the hormones of farm animals in order to improve their wellbeing is …	Οι ορμονικές αλλαγές στα αγροτικά ζώα για τη βελτίωση της ευζωίας τους είναι ...
4	Changing the hormone balance of farm animals to reduce costs without harming them is …	Η επιλεκτική αναπαραγωγή φυτών για τη βελτίωση των σπόρων για τη γεωργία είναι ...
5	Selectively breeding plants to improve crops for farming is…	Η μεταβολή της ορμονικής ισορροπίας των αγροτικών ζώων για τη μείωση του κόστους χωρίς να τα βλάπτει είναι ...
Answers were given on a 6-point Likert scale ranging from (1) “morally bad” to (6) “morally good”.		Οι απαντήσεις δόθηκαν σε 6βάθμια κλίμακα Likert, από (1) "ηθικά κακό" έως (6) "ηθικά καλό".
