# Supplementary material for: The genetic technologies questionnaire in the Greek-speaking population: the moral judgement of the lay public
Source: Front Genet. 2025 May 13;16:1594724. doi: 10.3389/fgene.2025.1594724 (PMC12106406; doi:10.3389/fgene.2025.1594724)
Supplement: Supplementary file 2 [file DataSheet4.pdf]

Measure of sample adequacy (anti-image correlation)

Item	MSA
1	0.932
2	0.916
3	0.929
4	0.902
5	0.865
6	0.895
7	0.926
8	0.935
9	0.89
10	0.909
11	0.884
12	0.945
13	0.866
14	0.858
15	0.945
16	0.958
17	0.958
18	0.924
19	0.913
20	0.946
21	0.955
22	0.944
23	0.956
24	0.947
25	0.961
26	0.948
27	0.955
28	0.93
29	0.979
30	0.929
