# Supplementary material for: The genetic technologies questionnaire in the Greek-speaking population: the moral judgement of the lay public
Source: Front Genet. 2025 May 13;16:1594724. doi: 10.3389/fgene.2025.1594724 (PMC12106406; doi:10.3389/fgene.2025.1594724)
Supplement: Supplementary file 3 [file DataSheet3.pdf]

		M	SD	item-total correlation	alpha if removed	Skewness	Standard Error of Skewness	Kurtosis
**GTQ30**	**GTQ_1**	5,25	1,13	0,25	0,930	-1,80	0,15	3,06
	**GTQ_2**	5,60	0,80	0,32	0,929	-2,45	0,15	7,09
	**GTQ_3**	5,24	1,15	0,39	0,929	-1,65	0,15	2,33
	**GTQ_4**	5,40	0,95	0,41	0,929	-1,92	0,15	4,19
	**GTQ_5**	4,77	1,39	0,37	0,929	-1,03	0,15	0,35
	**GTQ_6**	4,01	1,51	0,55	0,927	-0,31	0,15	-0,81
	**GTQ_7**	4,44	1,41	0,57	0,927	-0,63	0,15	-0,39
	**GTQ_8**	4,52	1,37	0,68	0,925	-0,71	0,15	-0,22
	**GTQ_9**	3,93	1,69	0,34	0,930	-0,38	0,15	-1,06
	**GTQ_10**	4,24	1,39	0,46	0,928	-0,46	0,15	-0,59
	**GTQ_11**	2,41	1,64	0,43	0,929	0,94	0,15	-0,33
	**GTQ_12**	5,00	1,15	0,34	0,929	-0,85	0,15	-0,38
	**GTQ_13**	2,38	1,55	0,35	0,929	0,90	0,15	-0,36
	**GTQ_14**	2,55	1,41	0,28	0,930	0,71	0,15	-0,28
	**GTQ_15**	3,41	1,58	0,56	0,927	0,02	0,15	-1,01
	**GTQ_16**	2,56	1,49	0,60	0,926	0,69	0,15	-0,45
	**GTQ_17**	4,09	1,55	0,57	0,927	-0,51	0,15	-0,71
	**GTQ_18**	3,76	1,54	0,65	0,926	-0,17	0,15	-0,93
	**GTQ_19**	3,31	1,68	0,63	0,926	0,15	0,15	-1,16
	**GTQ_20**	3,35	1,49	0,45	0,928	0,03	0,15	-0,86
	**GTQ_21**	4,20	1,30	0,48	0,928	-0,27	0,15	-0,47
	**GTQ_22**	3,04	1,52	0,64	0,926	0,26	0,15	-0,85
	**GTQ_23**	3,58	1,47	0,75	0,924	0,01	0,15	-0,79
	**GTQ_24**	3,11	1,48	0,69	0,925	0,26	0,15	-0,71
	**GTQ_25**	4,14	1,48	0,67	0,925	-0,41	0,15	-0,74
	**GTQ_26**	2,83	1,55	0,66	0,925	0,51	0,15	-0,72
	**GTQ_27**	3,44	1,62	0,55	0,927	0,05	0,15	-1,11
	**GTQ_28**	3,74	1,59	0,75	0,924	-0,21	0,15	-1,00

	**GTQ_29**	3,51	1,52	0,74	0,924	0,01	0,15	-0,91
	**GTQ_30**	3,75	1,56	0,73	0,924	-0,16	0,15	-0,96
**CTQ5**	**CTQ_1**	5,16	1,14	0,374	0,760	-1,42	0,15	1,69
	**CTQ_2**	3,70	1,64	0,482	0,733	-0,07	0,15	-1,11
	**CTQ_3**	3,37	1,51	0,664	0,661	0,15	0,15	-0,89
	**CTQ_4**	3,07	1,44	0,568	0,699	0,30	0,15	-0,67
	**CTQ_5**	3,93	1,54	0,551	0,705	-0,28	0,15	-0,90

Standard Error of Kurtosis	Standardized Skewness (z-skew)	Standardized Kurtosis (z-kurt)
0,31	-11,68	0,19
0,31	-15,92	13,21
0,31	-10,67	-2,18
0,31	-12,47	3,87
0,31	-6,68	-8,54
0,31	-2,00	-2,64
0,31	-4,06	-1,27
0,31	-4,58	-0,71
0,31	-2,49	-3,42
0,31	-3,01	-1,91
0,31	6,12	-1,08
0,31	-5,51	-1,26
0,31	5,86	-1,18
0,31	4,61	-0,92
0,31	0,13	-3,30
0,31	4,47	-1,47
0,31	-3,32	-2,05
0,31	-1,07	-3,05
0,31	0,95	-3,71
0,31	0,17	-2,80
0,31	-1,75	-1,53
0,31	1,67	-2,77
0,31	0,04	-2,58
0,31	1,71	-2,32
0,31	-2,65	-2,39
0,31	3,33	-2,34
0,31	0,31	-3,62
0,31	-1,37	-3,26

0,31	0,08	-3,02
0,31	-1,04	-12,89
0,31	-9,23	-4,27
0,31	-0,47	-13,38
0,31	0,97	-12,66
0,31	1,95	-11,97
0,31	-1,80	-12,70
