# Supplementary material for: The genetic technologies questionnaire in the Greek-speaking population: the moral judgement of the lay public
Source: Front Genet. 2025 May 13;16:1594724. doi: 10.3389/fgene.2025.1594724 (PMC12106406; doi:10.3389/fgene.2025.1594724)
Supplement: Supplementary file 4 [file DataSheet1.pdf]

STROBE Statement—Checklist of items that should be included in reports of ***cross-sectional studies***

	Item No	Recommendation	Page No
**Title and abstract**	1	(*a*) Indicate the study's design with a commonly used term in the title or the abstract	1-2
		(*b*) Provide in the abstract an informative and balanced summary of what was done and what was found	1-2
**Introduction**			
Background/rationale	2	Explain the scientific background and rationale for the investigation being reported	2-6
Objectives	3	State specific objectives, including any prespecified hypotheses	2-6
**Methods**			
Study design	4	Present key elements of study design early in the paper	6-12
Setting	5	Describe the setting, locations, and relevant dates, including periods of recruitment, exposure, follow-up, and data collection	6
Participants	6	(*a*) Give the eligibility criteria, and the sources and methods of selection of participants	6
Variables	7	Clearly define all outcomes, exposures, predictors, potential confounders, and effect modifiers. Give diagnostic criteria, if applicable	6-12
Data sources/ measurement	8*	For each variable of interest, give sources of data and details of methods of assessment (measurement). Describe comparability of assessment methods if there is more than one group	6-12
Bias	9	Describe any efforts to address potential sources of bias	9-11
Study size	10	Explain how the study size was arrived at	
Quantitative variables	11	Explain how quantitative variables were handled in the analyses. If applicable, describe which groupings were chosen and why	6-12
Statistical methods	12	(*a*) Describe all statistical methods, including those used to control for confounding	9-11
		(*b*) Describe any methods used to examine subgroups and interactions	9-11
		(*c*) Explain how missing data were addressed	9
		(*d*) If applicable, describe analytical methods taking account of sampling strategy	power analysis (p. 9-10), non-parametric tests for all analyses
		(*e*) Describe any sensitivity analyses	n.a., not a diagnostic instrument
**Results**			
Participants	13*	(a) Report numbers of individuals at each stage of study—eg numbers potentially eligible, examined for eligibility, confirmed eligible, included in the study, completing follow-up, and analysed	12
		(b) Give reasons for non-participation at each stage	n.a.

		(c) Consider use of a flow diagram	n.a.
Descriptive data	14*	(a) Give characteristics of study participants (eg demographic, clinical, social) and information on exposures and potential confounders	12-13
		(b) Indicate number of participants with missing data for each variable of interest	n.a., there were only full datasets
Outcome data	15*	Report numbers of outcome events or summary measures	12-26
Main results	16	(*a*) Give unadjusted estimates and, if applicable, confounder-adjusted estimates and their precision (eg, 95% confidence interval). Make clear which confounders were adjusted for and why they were included	12-26
		(*b*) Report category boundaries when continuous variables were categorized	12, table notes
		(*c*) If relevant, consider translating estimates of relative risk into absolute risk for a meaningful time period	n.a. there is no risk assessment
Other analyses	17	Report other analyses done—eg analyses of subgroups and interactions, and sensitivity analyses	12-26
**Discussion**			
Key results	18	Summarise key results with reference to study objectives	27
Limitations	19	Discuss limitations of the study, taking into account sources of potential bias or imprecision. Discuss both direction and magnitude of any potential bias	31
Interpretation	20	Give a cautious overall interpretation of results considering objectives, limitations, multiplicity of analyses, results from similar studies, and other relevant evidence	27-32
Generalisability	21	Discuss the generalisability (external validity) of the study results	27-32
**Other information**			
Funding	22	Give the source of funding and the role of the funders for the present study and, if applicable, for the original study on which the present article is based	33
