# Supplementary material for: The genetic technologies questionnaire in the Greek-speaking population: the moral judgement of the lay public
Source: Front Genet. 2025 May 13;16:1594724. doi: 10.3389/fgene.2025.1594724 (PMC12106406; doi:10.3389/fgene.2025.1594724)
Supplement: Supplementary file 5 [file DataSheet5.pdf]

PCA factor loadings for GTQ30

	Component 1	Component 2	Component 3	Component 4	Component 5
GTQ_30	0,87				
GTQ_28	0,84				
GTQ_26	0,78				
GTQ_25	0,77				
GTQ_24	0,75				
GTQ_23	0,71				
GTQ_29	0,70				
GTQ_27	0,46				
GTQ_19		0,70			
GTQ_18		0,69			
GTQ_17		0,64			
GTQ_21		0,55			
GTQ_22		0,55			
GTQ_20		0,47			
GTQ_1			0,71		
GTQ_4			0,71		
GTQ_3			0,71		
GTQ_2			0,67		
GTQ_10			0,60		
GTQ_12		0,43	0,59		
GTQ_13				0,80	
GTQ_11				0,79	
GTQ_14				0,62	
GTQ_9				0,53	
GTQ_15		0,42		0,52	
GTQ_16	0,36	0,35		0,50	
GTQ_5					0,78
GTQ_6					0,74
GTQ_7					0,71
GTQ_8					0,51

Explained variance

Component	Rotated sum of squared loadings		
	Total	% of Variance	Cumulative %
1	5.789	19.298	19.298
2	3.421	11.402	30.700
3	3.346	11.154	41.854
4	3.268	10.893	52.747
5	2.562	8.541	61.287
